# Protocol update for late endothelial progenitor cells identified by double‐positive staining

**DOI:** 10.1111/jcmm.17079

**Published:** 2021-12-14

**Authors:** Lishan Wu, Weijie Chen, Zeliang Chen, Jing Cao, Xiaoqing Dai, Hongjuan Chen, Xuerui Tan, Min Yu

**Affiliations:** ^1^ Department of Cardiology The First Affiliated Hospital Shantou University Medical College Shantou China; ^2^ Laboratory of Molecular Cardiology The First Affiliated Hospital Shantou University Medical College Shantou China; ^3^ The First Affiliated Hospital Shantou University Medical College Shantou China; ^4^ Department of Cardiology The First Affiliated Hospital of Henan University of Science and Technology Luoyang China

## Abstract

Endothelial progenitor cells (EPCs), which are precursors of endothelial cells (ECs), have the capacity to circulate, proliferate and differentiate into mature ECs. EPCs are primarily identified by the uptake of 1,1‐dioctadecyl‐3,3,3,3‐tetramethylindocarbocyanine‐labelled acetylated low‐density lipoprotein (Dil‐acLDL) and the binding of fluorescein‐isothiocyanate (FITC)‐conjugated Ulex europaeus agglutinin lectin (FITC‐UEA‐I). However, the cytoplasm and nucleus are usually stained by FITC‐UEA‐I via a typical method to double‐stain late EPCs. It is necessary to explore a new method to improve the quality of fluorescence photomicrographs of late EPCs stained with FITC‐UEA‐I. Here, we described an updated protocol for double‐staining late EPCs with Dil‐acLDL and FITC‐UEA‐I, with the cells more optimally stained with FITC‐UEA‐I.

## 1 INTRODUCTION

Endothelial progenitor cells (EPCs) are mixed cell populations that are mainly derived from peripheral blood, umbilical cord blood, bone marrow and tissue‐resident niches,^1^, ^2^, ^3^, ^4^ and these cells have the capacity to promote angiogenesis in ischaemic tissues. Since being described in 1997 by Asahara,^1^ EPCs have received a vast amount of research interest.

**FIGURE 1:**
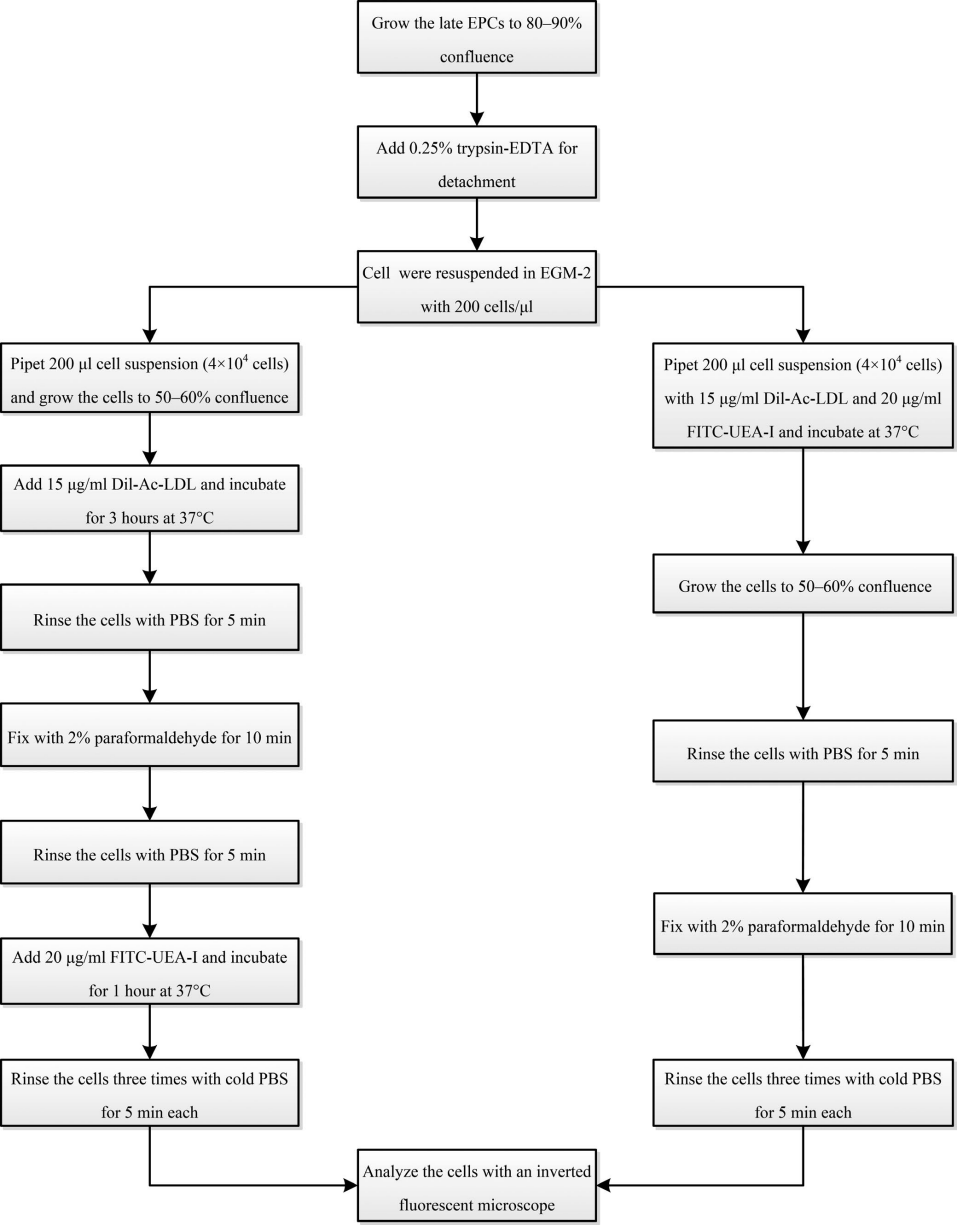
Protocol for late EPCs identification by double‐positive staining with Dil‐acLDL and FITC‐UEA‐I. Left, the typical protocol. Right, the updated protocol

According to the time at which they appear in culture, morphologies, growth patterns and function in vitro, EPCs can clearly be divided into two distinct populations: early and late EPCs.^5^, ^6^ These subpopulations were primarily identified by the uptake of 1,1‐dioctadecyl‐3,3,3,3‐tetramethylindocarbocyanine‐labelled acetylated low‐density lipoprotein (DiI‐acLDL) and the binding of fluorescein‐isothiocyanate (FITC)‐conjugated Ulex europaeus agglutinin lectin (FITC‐UEA‐I), as determined by fluorescence microscopy^7^, ^8^, ^9^, ^10^, ^11^; these cells were further shown to express endothelial cell (EC) markers such as von Willebrand factor (vWF), kinase insert domain receptor (KDR), CD105, CD146 and CD144 but not haematopoietic cell‐specific surface antigens, including CD11b, CD14 and CD45.^12^ Fluorescent staining was used to detect the uptake of DiI‐acLDL and the binding of FITC‐UEA‐I. Typically, the protocol for double‐staining EPCs^8^, ^9^, ^10^, ^11^ was as follows: Adherent cells were first incubated with DiI‐acLDL for 1–3 hours and were then incubated with FITC‐UEA‐I for 1 hour after being fixed for 10 min. Although the protocol was not complicated (Figure 1(left)), it is not easy to obtain good‐quality fluorescence photomicrographs of the binding of FITC‐UEA‐I to EPCs with an inverted fluorescence microscope. FITC‐UEA‐I usually binds to the cytoplasm, nucleus and cell debris. In our experience, less than 20% of the samples can be optimally stained with FITC‐UEA‐I. It is necessary to explore a new method to improve the quality of fluorescence photomicrographs of late EPCs stained with FITC‐UEA‐I.

Here, we describe an updated protocol for the uptake of DiI‐acLDL and the binding of FITC‐UEA‐I (Figure 1(right)). This method is simple and more efficient for obtaining good‐quality fluorescence photomicrographs of FITC‐UEA‐I binding than previous methods.

## 2 MATERIALS AND METHODS

### 2.1 Isolation and culture of late EPCs

The study was approved by the hospital Ethics Committee. Human umbilical cord blood samples (20–30 ml) were collected from healthy newborns with informed consent. Late EPCs were cultured according to previously described techniques.^3^, ^11^, ^12^ Briefly, total mononuclear cells (MNCs) were isolated from umbilical cord blood by Ficoll density gradient centrifugation. Cells were plated on six‐well plates and maintained in endothelial cell growth medium‐2 (EGM‐2; Lonza, Walkersville, MD, USA). After 24 h of culture, the medium was changed for the first time, then changed daily for 7 days and finally changed every other day until the first passage. Late EPCs at passages 3–5 were used for fluorescent staining.

### 2.2 Typical method for double‐staining late EPCs

The typical method for double‐staining late EPCs with DiI‐acLDL and FITC‐UEA‐I was performed according to techniques as previously described.^8^, ^9^, ^10^, ^11^ Late EPCs (4 × 10^4^ cells) in 200 µl of EGM‐2 were plated on 48‐well plates. When the cells grew to 50–60% confluence, new medium containing 15 μg/ml DiI‐acLDL was added and incubated for 3 h at 37 °C. Late EPCs were washed with phosphate‐buffered saline (PBS), fixed for 10 min with 2% paraformaldehyde, washed again for 5 min with PBS and then incubated with 20 μg/ml FITC‐UEA‐I for 1 h. After being washed 3 times with PBS for 5 min each, late EPCs were observed under an inverted fluorescence microscope.

### 2.3 Staining late EPCs with FITC‐UEA‐I after paraformaldehyde fixation

Late EPCs were plated on 48‐well plates. After the cells had grown to 50–60% confluence, the cells were washed with PBS, fixed with 2% paraformaldehyde for 10 min, washed again for 5 min with PBS and then incubated with 20 μg/ml FITC‐UEA‐I for 1 h. After being washed with PBS, the cells were observed.

### 2.4 Staining late EPCs with FITC‐UEA‐I before paraformaldehyde fixation

Late EPCs and 20 μg/ml FITC‐UEA‐I were plated on 48‐well plates. The cells were washed with PBS and fixed with paraformaldehyde when they reached 50–60% confluence. Then, the cells were washed again with PBS and observed.

### 2.5 Updated protocol for double‐staining late EPCs

Cell suspensions containing late EPCs, 15 μg/ml DiI‐acLDL and 20 μg/ml FITC‐UEA‐I were seeded on 48‐well plates. After the cells had grown to 50–60% confluence, late EPCs were washed with PBS and fixed with 2% paraformaldehyde for 10 min. After being washed again with PBS, late EPCs were analysed with a fluorescence microscope.

### 2.6 Evaluation of immunofluorescence images of EPCs stained with FITC‐UEA‐I

Sixty‐four samples of double‐stained late EPCs were included in the present study. A total of 34 and 30 samples were stained with the typical and updated protocols respectively. An optimally stained immunofluorescence image was defined as binding of FITC‐UEA‐I to the cell membrane, no staining of the cytoplasm, nucleus, or cell debris and low background staining. The results were evaluated by two independent reviewers in a blinded manner.

### 2.7 Statistical analysis

The data are presented as the number of samples. Differences between group means were assessed by chi‐square tests using SPSS 24.0. *p* values <0.05 were considered significant.

## 3 RESULTS

**FIGURE 2:**
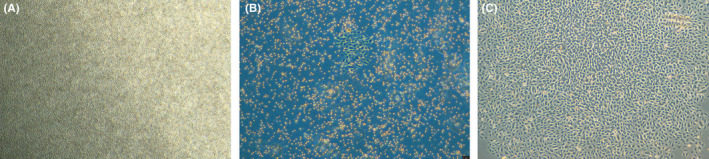
Morphology of mononuclear cells and late EPCs. Mononuclear cells had a round morphology after being isolated from human umbilical cord blood (A). Late EPCs appeared early (approximately one week) as small colonies after culture (B), and late EPCs had a cobblestone‐like cell morphology (C)

**FIGURE 3:**
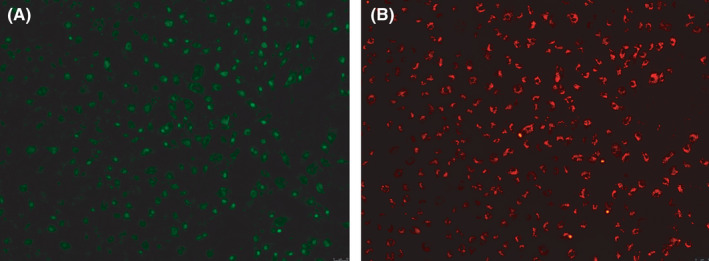
Late EPCs were stained by the typical protocol. Although late EPCs were positive for both FITC‐UEA‐I (A) and DiI‐acLDL (B), FITC‐UEA‐I bound to the cytoplasm and nucleus in addition to the membrane (A), which made it difficult to obtain good‐quality fluorescence photomicrographs of late EPCs

**FIGURE 4:**
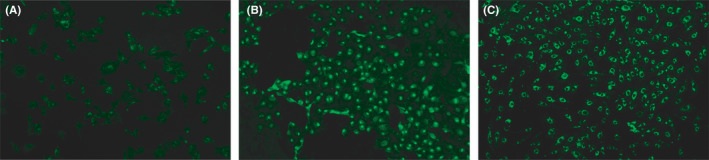
Staining of late EPCs with FITC‐UEA‐I after and before paraformaldehyde fixation. FITC‐UEA‐I usually binds to cell debris, the cytoplasm and the nucleus because of the destruction of cellular architecture by paraformaldehyde fixation when the cells were incubated with FITC‐UEA‐I after paraformaldehyde fixation (A, B). When late EPCs were incubated with FITC‐UEA‐I before fixation, the binding of FITC‐UEA‐I to the cytoplasm and nucleus was prevented (C)

**FIGURE 5:**
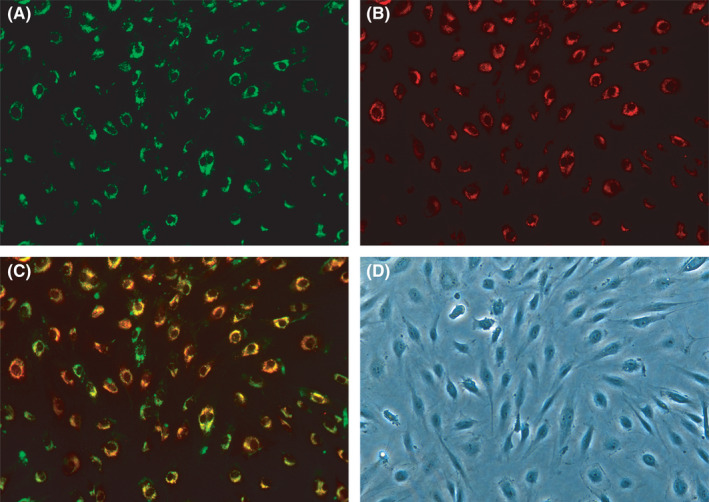
Late EPCs were positive for both DiI‐acLDL (A) and FITC‐UEA‐I (B) with the updated protocol. Merged image of both stains (C) and an image of the light micrograph (D, 200×)

MNCs had a round morphology after being isolated from human umbilical cord blood and plated on six‐well plates (Figure 2A). After 4–5 days in culture, the nonattached cells are removed and the adherent cells (early EPCs) appeared to be clusters, with a spindle shape. They gradually disappeared in 4 weeks.^5^ Late EPCs appeared early, at approximately one week, and formed small colonies after culture (Figure 2B) and later developed a cobblestone‐like cell morphology (Figure 2C). Fluorescence microscopy showed that late EPCs were double positive for FITC‐UEA‐1 and DiI‐acLDL by a typical method (Figure 3A,B). Although the typical method of double‐staining late EPCs has been used for over twenty years, staining with FITC‐UEA‐I was not optimal, and the cytoplasm and nucleus (Figure 3A) were both stained, which may be associated with destruction of the cell structure by paraformaldehyde fixation. To investigate the effects of paraformaldehyde fixation on the binding of FITC‐UEA‐I to late EPCs, the cells were incubated with FITC‐UEA‐I before or after paraformaldehyde fixation. The results showed that FITC‐UEA‐I could bind to the cell membrane, cytoplasm and nucleus when the cells were incubated with FITC‐UEA‐I after paraformaldehyde fixation (Figure 4A,B). When late EPCs were incubated with FITC‐UEA‐I before fixation, the binding of FITC‐UEA‐I to the cytoplasm and nucleus was prevented (Figure 4C). Based on these results, we updated the protocol for double‐staining late EPCs (Figure 1 (right)). In the present study, of 34 samples, only 6 samples (17.6%) were optimally stained with FITC‐UEA‐I (Table 1) by the typical protocol. After using the updated protocol, 28 of 30 samples (93.3%) were optimally stained (Table 1) with FITC‐UEA‐I, with one sample failing to be stained and one sample that was not stained because of cell pollution. It is very easy to obtain good‐quality fluorescence photomicrographs of late EPCs with this updated protocol (Figure 5).

**TABLE 1:** Optimally stained immunofluorescence image of late EPCs with the typical and updated protocols

	Optimally stained immunofluorescence images of late EPCs with FITC‐UEA‐I		Chi‐square result	*p* value
	+	−		
Typical protocol	6	28	36.66	0.0000
Updated protocol	28	2		

## 4 DISCUSSION

During the past twenty years, EPCs have been widely investigated, including the effects of various risk factors on EPCs,^13^ the relationship between EPCs and disease outcomes^14^ and EPC‐based therapies.^15^ As of 1 September 2021, approximately 12,100 papers had been published in the PubMed database using the search term "endothelial progenitor cell.". The isolation and identification of EPCs is the first step in further research.

Two different types of EPCs have been recognized according to their time‐dependent appearance: early and late EPCs.^5^, ^6^ Early EPCs have a spindle shape, peak growth at 2 to 3 weeks, die at 4 weeks and contribute to neovasculogenesis thought paracrine effects, while late EPCs appear at 2 to 4 weeks (approximately one week, occasionally 3 days after MNCs were plated in our study) and provide a sufficient number of endothelial cells (ECs).^5^ Late EPCs represent actual endothelial progenitors. Therefore, the identification of late EPCs is essential for late EPC‐based therapies after the isolation of MNCs. Late EPCs are characterized by their morphologic appearance, acLDL uptake, lectin binding and the expression of KDR, vWF and CD markers of ECs.

AcLDL is taken up by ECs and can be used to isolate and identify ECs.^16^ UEA‐I binds to specific ECs and is regarded as a sensitive marker of ECs.^17^ Therefore, dual binding of DiI‐acLDL and FITC‐UEA‐I is usually used for the primary characterization of EPCs. Cells showing double‐positive fluorescence are identified as differentiating EPCs.^8^ However, in our experience, the cytoplasm and nucleus are usually stained by FITC‐UEA‐I, as viewed under an inverted fluorescence microscope with the typical method (Figure 3A), which makes it difficult to obtain good‐quality fluorescence photomicrographs of FITC‐UEA‐I.

Lectins are carbohydrate‐binding proteins that can bind to specific carbohydrate residues, most of which are derived from plant species, invertebrates and higher animals.^18^ UEA‐I, a lectin isolated from Ulex europaeus, shows specific affinity for certain 1‐fucose moieties and is a specific and sensitive marker for ECs.^17^ It has been reported that UEA‐I can bind to the cell membrane, cytoplasm and nucleus.^19^, ^20^

Fixation is an important step that is required for optimal immunofluorescence imaging. Paraformaldehyde fixation is commonly used. Although paraformaldehyde fixation results in the chemical crosslinking of free amino groups and helps to preserve cellular architecture, it can also damage cellular architecture. Incubation with FITC‐UEA‐I after fixation leads to the binding of FITC‐UEA‐I to the cytoplasm and nucleus, which may be the main cause of cytoplasmic and nuclear staining. To verify this hypothesis, late EPCs were incubated with FITC‐UEA‐I after and before paraformaldehyde fixation. The results showed that incubation after paraformaldehyde fixation led to the binding of FITC‐UEA‐I to the cytoplasm and nucleus (Figure 4A,B), which was prevented by incubation before paraformaldehyde fixation (Figure 4C).

Based on these results, we adjusted the protocol (Figure 1 (right)), and late EPCs were incubated with FITC‐UEA‐I before paraformaldehyde fixation. We found that when late EPCs were seeded onto 48‐well plates in the presence of 15 μg/ml DiI‐acLDL and 20 μg/ml FITC‐UEA‐I, washed with PBS and fixed with paraformaldehyde when the cells reached 50–60% confluence, it was easier to obtain good‐quality fluorescence photomicrographs (Figure 5).

Compared with previous methods, there are many advantages of this updated protocol. First, it is easier than typical method. When using this protocol, 200 μl of the cell suspension (4×10^4^ cells) was seeded on a 48‐well plate with 15 μg/ml DiI‐acLDL and 20 μg/ml FITC‐UEA‐I and incubated at 37 °C. After the cells had grown to 50–60% confluence, the cells were washed with PBS and fixed with 2% paraformaldehyde for 10 min. Then, the cells were washed again with PBS and observed under an inverted fluorescence microscope. Second, the updated method saves time; after the cells reach 50–60% confluence, the process (wash for 20 min and fixation for 10 min) can be completed within 30 min, while the previous method requires approximately 4.5 h (incubation with DiI‐acLDL for 3 h and FITC‐UEA‐I for 1 h, wash for 25 min and fixation for 10 min). A potential disadvantage of this protocol is that the time at which the cells were incubated with DiI‐acLDL and FITC‐UEA‐I was longer, requiring approximately 12–16 h, which can increase the risk of cell contamination.

In a series of experiments, 34 and 30 samples were stained with the typical and updated protocols, and 17.6% and 93.3% of the samples were optimally stained with FITC‐UEA‐I, respectively. Obtaining good‐quality fluorescence photomicrographs is easier with the updated protocol than with the typical method. Our data provide a promising double‐staining protocol with DiI‐acLDL and FITC‐UEA‐I for the primary characterization of late EPCs.

## CONFLICT OF INTEREST

There are no conflicts of interest.

## AUTHOR CONTRIBUTION

**Lishan Wu:** Writing – original draft (equal). **Weijie Chen:** Writing – original draft (equal). **Zeliang Chen:** Investigation (equal); Methodology (equal). **Jing Cao:** Investigation (equal); Methodology (equal). **Xiaoqing Dai:** Investigation (equal); Methodology (equal). **Hongjuan Chen:** Methodology (equal); Supervision (equal). **Xuerui Tan:** Writing – review & editing (equal). **Min Yu:** Conceptualization (equal).
